# Understanding trimester-specific miscarriage risk in Indian women: insights from the calendar data of National Family Health Survey (NFHS-5) 2019-21

**DOI:** 10.1186/s12905-023-02838-7

**Published:** 2024-01-23

**Authors:** Milan Das, Hemant Patidar, Mayank Singh

**Affiliations:** 1 International Institute for Population Sciences, Mumbai, India; 2 Dr Harisingh Gour Vishwavidyalaya, Sagar (M.P), India

**Keywords:** Miscarriage, Calendar data, Socioeconomic factors, Reproductive health, India

## Abstract

**Background:**

The primary public health issue, especially in low- and middle-income countries, is early pregnancy loss driven by miscarriage. Understanding early pregnancy losses and the characteristics of mothers who have miscarriages is essential to creating effective reproductive health strategies. Thus, this study’s primary goal is to delve into the factors which impact miscarriages that take place prior to and following the first 12 weeks of gestation.

**Methods:**

The bivariate analysis was employed to determine the frequency of miscarriages. The factors associated with miscarriages in the first (≤12 weeks) and second & above (> 12 weeks) trimesters of pregnancy were then examined using a generalised linear regression model, with 95% confidence intervals. Finally, we use ArcGIS to illustrate the prevalence of miscarriage in the districts of India.

**Results:**

Our result shows that miscarriages occur often in India (4.9%), with 23% of cases occurring in the first trimester (≤12 weeks). In our bivariate analysis, we identified several factors associated with a higher prevalence of miscarriages in India. It was found that mothers aged thirty years or older, residing in urban areas, with less than ten years of education, belonging to the richest wealth quantile, expressing a desire for more children, having no demand for contraception, and possessing no parity experienced a higher prevalence of miscarriage in total pregnancies in India. On the other hand, the generalised linear model’s findings show that mothers who are thirty years of age or older, practise other religions, live in urban areas, are members of other castes, want more children, marry before the age of eighteen, and meet their contraceptive needs are more likely to have miscarriages in total pregnancy. However, there is a larger likelihood of miscarriage in the first trimester (≤12 weeks) for mothers who follow other religions, live in urban areas, are from Other Backward Class (OBC), get married before the age of eighteen, and fall into the middle and upper wealth quantiles. A mother is more likely to miscarriage in the second & above (> 12 weeks) trimesters if she is older than thirty, from other castes, wants more children, has moderate media exposure, marries before turning eighteen, meets her contraceptive needs, and does not feel the need for contraception. After accounting for socioeconomic characteristics, all results were statistically significant.

**Conclusions:**

Given the substantial number of miscarriages in India, police need to improve planning and guidance in order to lower pregnancy loss due to miscarriage. Miscarriage rates may be significantly decreased by enhancing the availability and quality of reproductive health care infrastructure, particularly in rural areas.

**Supplementary Information:**

The online version contains supplementary material available at 10.1186/s12905-023-02838-7.

## Background

In low- and middle-income countries, there is an urgent requirement to promote better maternal health and well-being. However, adverse pregnancy outcomes, such as miscarriage, can negatively impact the well-being of the mother [1]. Miscarriage is a common detrimental pregnancy outcome affecting many mothers across the world.

In accordance with data from the World Health Organisation (WHO), miscarriages contribute to ten to fifteen per cent of all clinically confirmed pregnancies. Pregnancy loss has become widespread in low- and middle-income countries, according to the WHO [2]. Although early miscarriages frequently go overlooked, the actual rate may be substantially greater in low- and middle-income countries. According to recent data from the National Family Health Survey (NFHS), which has been carried out between 2019–21 in India, miscarriages occur in approximately 7% of pregnancies [3]. The prevalence of diseases and fatalities among women of reproductive age can be significantly affected by pregnancy loss, especially when it culminates in a miscarriage [4]. Pregnancy can often be terminated promptly by miscarriage, which is generally considered to be an early pregnancy loss that happens before the 20th week of gestation [5]. Remarkably, a substantial proportion of miscarriages—roughly 15% of pregnancies with medical confirmation occur before women are even cognizant that they are pregnant [6]. A miscarriage may result in a number of challenges, such as haemorrhage, sepsis, retained products, and disseminated intravascular coagulation (DIC), which can cause multiple organ failure [7].

The likelihood of miscarriage is determined by a wide range of socioeconomic factors, notably parity, age of the mother, employment, and exposure to reproductive healthcare services [8]. In addition, co-morbidities, infections, uterine dysfunction, mother age, genetic variations, hormone imbalances, and lifestyle choices are physiological factors that significantly lead to pregnancy loss [9]. Numerous factors, namely genetics, anatomical issues, endocrine disorders, thrombophilia, and viral infections, have been associated with miscarriages, according to studies [10]. The age and educational level of the mother are two variables which increase the likelihood of miscarriage [11]. As pointed out by [12], the risk is determined by an assortment of physiological and sociodemographic factors. Pregnancy loss, which includes stillbirth and miscarriage, affects the morbidity and mortality of women of reproductive age and faces major obstacles in accomplishing the Sustainable Development Goals (SDGs) [13]. Lower rates of miscarriage have been associated with women’s dietary practises that include antioxidants like zinc, selenium, and vitamins E and C, as well as diets high in vitamins B12 and D [14].

Indian women who are pregnant for the first time between the ages of 31–49 may be more likely to suffer than those who give birth earlier to spontaneous miscarriage, according to a study [15]. In addition, a population-based study carried out in India found that 44 out of 1000 women between the ages of 15–49 had miscarriages [6]. The likelihood of miscarriage jumps dramatically with age, from 10% for women in their 20–24 to 51% for those in their 40–44 [16]. An increased risk of unintended births has also been attributed to intimate partner abuse, which may result in additional miscarriages [17, 18]. According to a recent study, everyday difficulties, family pressure to have fewer children, and college degrees were all associated with pregnancy loss among Indian women. The northern and north-eastern parts of India were found to have higher rates of pregnancy loss and unplanned pregnancies, which can result in pregnancy loss or unwanted kids [19]. According to a study carried out in rural Maharashtra, 26% of pregnancy loss happened in the second trimester, possibly as a result of inadequate nutrition and sterilisation failures made during delayed pregnancy confirmation [20, 21]. Genetic causes are mainly to blame for spontaneous miscarriages; they are also not often responsible for recurrent miscarriages. There is debate regarding the connection between diseases like thyroid autoimmunity, subclinical thyroid problems, and miscarriage, particularly in cases of early miscarriage [22]. Additionally, a large number of literature also found that the women’s parity or calendar period is the risk factor for miscarriage [23–26]. Furthermore, a spectrum of research has shown that a woman’s parity or calendar period enhances her likelihood of miscarriage [23–26]. Research has indicated that consumption of alcohol and being an older mother constitute significant risk factors for miscarriage [27–30].

Despite the fact that the most of research indicates that miscarriages usually occur in the first trimester [31–36]. There is still a significant number of miscarriages in the second trimester, reaching an average of 44 per thousand pregnancies during various gestational stages [37]. Additionally, a couple of studies have looked at India’s miscarriage rates per trimester. Therefore, this study mainly bridges that gap by examining the prevalence of miscarriages in the first (≤12 weeks) and second & above (> 12 weeks) trimesters as well as related risk factors, with a focus on the country. We used the most present National Family Health Survey (NFHS) calendar data for 2019–21 in order to evaluate factors associated with miscarriage among Indian women aged 15–49.

## Methods

### Data source

We used data from the National Family Health Survey (NFHS-5), a nationally representative household survey carried out in India between 2019–21. The survey was conducted in all Indian states, Union Territories (UTs) and districts. NFHS-5 have been conducted under the stewardship of the Ministry of Health and Family Welfare (MoHFW), Government of India. MoHFW designated the International Institute for Population Sciences (IIPS), Mumbai, as the nodal agency for the NFHS-5. Funding for NFHS-5 was provided by the MoHFW, Government of India. ICF, USA provided technical assistance through the Demographic and Health Surveys (DHS) Program, which is funded by USAID. The CAB component of the NFHS-5 looked for to estimate the prevalence of malnutrition, anemia, hypertension, high blood glucose levels, waist and hip circumference, vitamin D3, hemoglobin A1c, and malaria parasites through biomarker tests and measurements. The NFHS-5 collected calendar data for the previous five years’ incidences of pregnancy, childbirth, contraceptive use, and discontinuation. The survey included 724,115 Indian women between the ages of 15–49.

### Sample selection

**Fig. 1:**
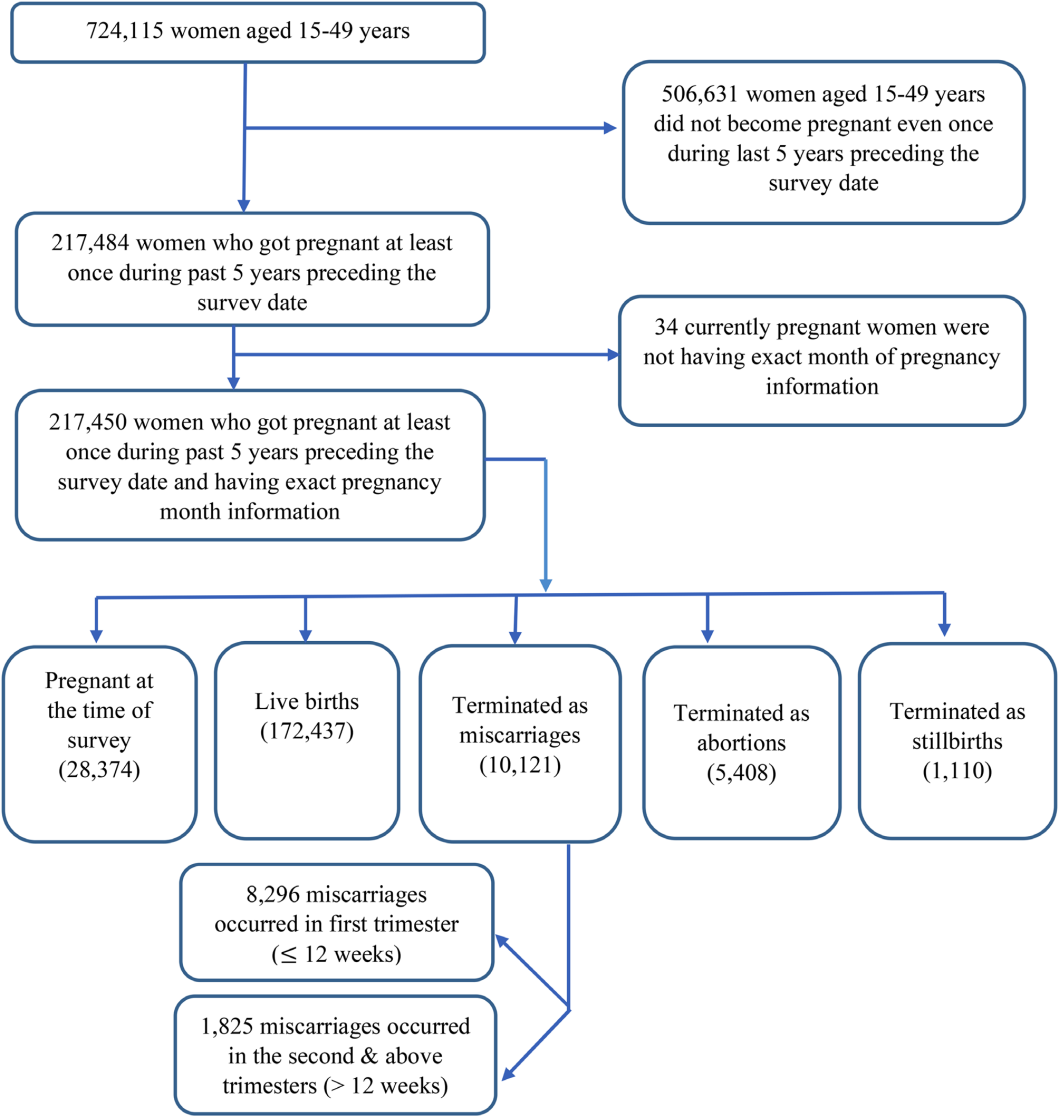
Flowchart for determining the final analytical study sample in the first trimester (≤12 weeks) and second & above trimesters (>12 weeks) in India, NFHS-2019–21 (Source: National Family Health Survey (NFHS) 2019-21, India)

In our study, we focused on a group of women who had experienced at least one pregnancy within the past five years, resulting in a total of 217,484 individuals (as shown in Fig. 1). In order, to streamline our analysis, we exclusively considered data from their most recent pregnancy outcome and excluded any outcomes from any previous pregnancies. Additionally, we excluded women with unclear pregnancy outcomes, leaving us with a final sample size of 217,450 respondents, each with well-defined pregnancy outcomes. These outcomes were categorized into distinct groups, which included current pregnancies (28,374), live births (172,437), miscarriages (10,121), abortions (5,408), or stillbirths (1,110). Out of the 10,121 miscarriages, 8,296 occurred in the first trimester (≤12 weeks), and 1,825 occurred in the second & above trimesters (>12 weeks). Figure 1 provides a more detailed explanation of our sample selection process for this study.

### Dependent variables

Our primary dependent variable in this study was the rate of miscarriage among female respondents (aged 15–49) with at least one pregnancy in the five years prior to the survey. Our strategy for assessing the occurrence of miscarriage consisted of employing a binary variable. Miscarriages that occurred in the first trimester (≤12 weeks) and the second & above trimesters (> 12 weeks) were classified as yes = 1 and no = 0, respectively.

### Independent variables

The selection of predictor variables for this study was guided by a rigorous review of the available literature [12, 38]. We considered several other independent variables in the analysis including the age groups of women (i.e., < 20 years, 20–29 years, 30 years and above); marital status (i.e., not in union, currently married); religious affiliation (i.e., Hindu, Muslims, and Others); place of residence (i.e., urban and rural); social class (i.e., Scheduled Castes (SCs), Scheduled Tribes (STs), Other Backward Classes (OBCs) and Other castes included forward caste); educational attainment of women (i.e., no education, ≤10 years, and > 10 years); household’s wealth index (i.e., poorest, poorer, middle, richer and richest); desire of children (yes, undecided or no more, and sterilised/infecund); mass media exposure (i.e., low, moderate and high); age at marriage (i.e.,<18 years, 18–24 years, and ≥25 years); contraceptive demand (i.e., unmet need, met need, and no demand), number of living children’s (i.e., no child, 1–3 child, 4 and above children); sex of the last child (male, female); geographical regions of India classified into six regions (i.e. East, West, North, South, Central, and North-east).

### Statistical analysis

In this study, we employed a series of statistical techniques to analyses the determinants of miscarriage in India based on various socio-demographic factors and gestational trimesters. First, we conducted a descriptive analysis to estimate the prevalence of miscarriage. Next, we used the prevalence map of miscarriage in the districts of India. Finally, we developed a generalized linear model with a binomial distribution assumption and a log link for the binary outcomes to determine the risk of miscarriage in response to specific socio-demographic variables. We also evaluated and assessed the multi-collinearity among independent variables using the variance inflation factor (VIF). The model output provided an adjusted odds ratio of miscarriage among reproductive-aged women and their corresponding 95% confidence interval (95% CI), with a significant level set at *p* < 0.05. For our analysis, we utilized Stata Software (version 16.0) and ArcGIS (version 10.3) for geospatial mapping.

## Results

Table 1 presents the demographic and socioeconomic characteristics of the study sample, consisting of women who experienced miscarriage in both the first (≤12 weeks) and second & above (> 12 weeks) trimesters, along with the distribution of overall pregnancies. Most samples, around 62.8%, fell in the age range of 20–29 years, identified as Hindu 73.4% and lived in rural areas 78%. Additionally, a significant proportion of samples, 37.7%, belonged to the Other Backward Classes (OBC) groups. In terms of education, the majority of the sample, 50.7%, had received one to ten years or less of education. Approximately 43.5% of participants expressed no desire for further children, and nearly one-third or 31.5% were married before reaching the age of 18. Lastly, 24.4% of samples were from the central region of India.

**Table 1:** Sample characteristics of miscarriage in reproductive-aged women for their last pregnancy in the first (≤12 weeks) and second & above (> 12 weeks) trimesters by selected socio-demographic characteristics in India, NFHS, 2019-21

Variables	Total Pregnancy	Miscarriage	
		First trimester (≤12 weeks)	Second & above trimesters (> 12 weeks)
**Age group**			
<20 years	7,970 (3.7)	3,52 (82.8)	73 (17.2)
20–29 years	13,6512 (62.8)	4,560 (82.3)	9,83 (17.7)
≥30 years	72,968 (33.6)	3,384 (81.5)	7,69 (18.5)
**Marital status**			
Not in union	3,844 (1.8)	1,57 (79.7)	40 (20.3)
Currently married	213,606 (98.2)	8,139 (82)	1,785 (18)
**Religion**			
Hindu	159,668 (73.4)	6,311 (81.9)	1,391 (18.1)
Muslim	31,075 (14.3)	1,070 (80.9)	2,52 (19.1)
Others	26,707 (12.3)	9,15 (83.4)	1,82 (16.6)
**Place of residence**			
Urban	47,828 (22)	2,250 (85.2)	3,91 (14.8)
Rural	169,622 (78)	6,046 (80.8)	1,434 (19.2)
**Caste**			
Scheduled Castes (SCs)	42,981 (19.8)	1,709 (81.0)	4,00 (19.0)
Scheduled Tribes (STs)	43,354 (19.9)	1,261 (78.8)	3,39 (21.2)
Other Backward Class (OBC)	81,975 (37.7)	3,248 (82.8)	6,74 (17.2)
Others	49,140 (22.6)	2,078 (83.5)	4,12 (16.6)
**Education in years**			
No education	43,560 (20.0)	1,444 (78.0)	4,07 (22.0)
≤10 years	110,235 (50.7)	4,272 (81.2)	9,92 (18.8)
>10 years	63,655 (29.3)	2,580 (85.8)	4,26 (14.2)
**Wealth Index**			
Poorest	53,492 (24.6)	1,593 (77)	4,77 (23)
Poorer	49,367 (22.7)	1,793 (80.7)	4,29 (19.3)
Middle	42,949 (19.8)	1,753 (81.8)	3,89 (18.2)
Richer	38,808 (17.9)	1,618 (83.8)	3,13 (16.2)
Richest	32,834 (15.1)	1,539 (87.6)	2,17 (12.4)
**Desire of children**			
Yes	83,742 (38.7)	4,691 (81)	1,103 (19)
Undecided or no more	94,152 (43.5)	2,744 (84.8)	4,92 (15.2)
Sterilised/Infecund	38,447 (17.8)	7,80 (78.6)	2,13 (21.5)
**Mass media exposure**			
Low	145,044 (66.7)	5,228 (81.2)	1,212 (18.8)
Moderate	49,870 (22.9)	2,119 (82.9)	4,36 (17.1)
High	22,536 (10.4)	9,49 (84.3)	1,77 (15.7)
**Age at marriage**			
<18 years	67,572 (31.5)	2,542 (80.0)	6,37 (20.0)
18–24 years	124,238 (57.9)	4638 (83.0)	9,52 (17.0)
≥25 years	22,785 (10.6)	1,014 (82.3)	2,18 (17.7)
**Contraceptive demand**			
Unmet need	35,090 (16.2)	9,71 (84.4)	1,80 (15.6)
Met need	119,583 (55.1)	3,743 (83.3)	7,48 (16.7)
No demand	62,293 (28.7)	3,555 (79.9)	8,92 (20.1)
**Number of living children**			
No children	17,108 (7.9)	2,486 (80.2)	6,13 (19.8)
1–3 children	175,936 (80.9)	5,180 (82.7)	1,082 (17.3)
4 and above children	24,406 (11.2)	6,30 (82.9)	1,30 (17.1)
**Sex of the last child**			
Male	109,318 (54.1)	3,209 (83.2)	6,19 (16.2)
Female	92,719 (45.9)	2,687 (81.0)	6,29 (19.0)
**Region**			
East	40,648 (18.7)	1,588 (81.1)	3,71 (18.9)
West	19,383 (8.9)	6,11 (80.8)	1,45 (19.2)
North	41,351 (19.0)	1,866 (84.4)	3,44 (15.6)
South	28,449 (13.1)	8,69 (79.6)	2,23 (20.4)
Central	53,001 (24.4)	2,226 (82.2)	4,82 (17.8)
Northeast	34,618 (15.9)	1,136 (81.4)	2,60 (18.6)
**Total**	**217,450 (100)**	**8,296 (82)**	**1,825 (18)**

Source: National Family Health Survey (NFHS) 2019-21, India

The study primarily focuses on the trimester-specific incidence of miscarriage among pregnant women in the years preceding the survey. Among the sample of miscarriages, 82.8% occurred among women aged < 20 years within the first trimester, with the remaining 17.2% of the sample experiencing miscarriages after 12 weeks of gestation (> 12 weeks). Among currently married women, 82% of miscarriages took place within the first 12 weeks, while 20.3% of miscarriages among unmarried women occurred after the first trimester (> 12 weeks). Overall, the sample indicates that a higher proportion of miscarriages occurred during the first trimester across various background characteristics of women. Approximately one-fifth of miscarriages happened during the second and subsequent trimesters, regardless of the women’s background characteristics.

Table 2 presents the prevalence of miscarriage among women of reproductive age during the first (≤12 weeks) and second & above trimesters (> 12 weeks) trimesters. The results reveal that the overall prevalence of miscarriage was higher among the following groups: unmarried women (6%), urban residents (5.7%), women with ten years or less of education (5.1%), the wealthiest women (5.4%), those who desire more children (7.6%), and women with no living children (19.2%). In northern (5.6%) and central (5.6%) regions, the prevalence of miscarriage was higher than in other regions.

**Table 2:** Prevalence of miscarriage in the first (≤12 weeks) and second & above (>12 weeks) trimesters among women of reproductive age for their last pregnancy in India, NFHS, 2019-21

Variables	Miscarriage		
	Total Pregnancy	First trimester (≤12 weeks)	Second & above trimesters (> 12 weeks)
**Age group**			
<20 years	5.8 [5.0, 6.5]	26.3 [22.9, 29.7]	1.3 [1.0, 1.7]
20–29 years	4.3 [4.2, 4.5]	25.2 [24.3, 26.1]	0.9 [0.8, 0.9]
≥30 years	6.0 [5.7, 6.2]	20.3 [19.4, 21.2]	1.4 [1.3, 1.6]
**Marital status**			
Not in union	6.0 [4.8, 7.2]	19.5 [15.6, 23.4]	1.7 [0.8, 2.7]
Currently married	4.9 [4.8, 5.0]	23.2 [22.6, 23.9]	1.0 [1.0, 1.1]
**Religion**			
Hindu	5.0 [4.9, 5.2]	23.3 [22.6, 24]	1.1 [1.0, 1.2]
Muslim	4.4 [4.0, 4.7]	22.3 [20.6, 24.1]	1.0 [0.8, 1.1]
Others	4.8 [4.2, 5.4]	22.8 [19.9, 25.6]	1.0 [0.6, 1.3]
**Place of residence**			
Urban	5.7 [5.3, 6.0]	25.3 [23.9, 26.6]	1.0 [0.9, 1.2]
Rural	4.6 [4.5, 4.7]	22.2 [21.5, 22.8]	1.1 [1.0, 1.1]
**Caste**			
Scheduled Castes (SCs)	5.1 [4.8, 5.4]	23.3 [22.1, 24.6]	1.2 [1.0, 1.4]
Scheduled Tribes (STs)	4.2 [3.8, 4.6]	21.5 [19.5, 23.6]	1.0 [0.8, 1.2]
Other Backword Class (OBC)	4.8 [4.6, 5.0]	23.2 [22.3, 24.1]	1.0 [0.9, 1.1]
Others	5.2 [4.9, 5.5]	23.5 [22.2, 24.8]	1.0 [0.9, 1.1]
**Education in years**			
No education	4.7 [4.4, 5.0]	20.5 [19.3, 21.8]	1.4 [1.2, 1.6]
≤10 years	5.1 [4.9, 5.2]	22.9 [22.1, 23.8]	1.1 [1.0, 1.2]
>10 years	4.8 [4.5, 5.0]	25.2 [24.0, 26.5]	0.7 [0.6, 0.9]
**Wealth Index**			
Poorest	4.1 [3.9, 4.4]	20.1 [18.9, 21.3]	1.2 [1.0, 1.3]
Poorer	4.8 [4.5, 5.0]	23.0 [21.8, 24.3]	1.1 [0.9, 1.2]
Middle	5.3 [5.0, 5.6]	24.0 [22.7, 25.3]	1.2 [1.0, 1.4]
Richer	5.1 [4.8, 5.4]	23.7 [22.3, 25.1]	0.9 [0.8, 1.1]
Richest	5.4 [5.1, 5.8]	24.9 [23.3, 26.4]	0.8 [0.7, 1.0]
**Desire of children**			
Yes	7.6 [7.3, 7.8]	36.9 [35.7, 38.2]	1.7 [1.6, 1.8]
Undecided or no more	3.6 [3.5, 3.8]	17.9 [17.0, 18.7]	0.7 [0.6, 0.7]
Sterilized/Infecund	2.4 [2.2, 2.6]	9.9 [9.0, 10.8]	0.7 [0.5, 0.8]
**Mass media exposure**			
Low	4.8 [4.6, 4.9]	23 [22.3, 23.7]	1.1 [1.0, 1.1]
Moderate	5.1 [4.8, 5.4]	23.1 [21.8, 24.4]	1.0 [0.9, 1.2]
High	5.3 [4.9, 5.8]	24.0 [22, 26.1]	1.0 [0.8, 1.3]
**Age at marriage**			
<18 years	5.0 [4.8, 5.3]	21.9 [20.9, 22.9]	1.2 [1.1, 1.4]
18–24 years	4.7 [4.5, 4.9]	23.5 [22.7, 24.3]	0.9 [0.9, 1.0]
≥25 years	5.7 [5.3, 6.2]	26.3 [24.1, 28.4]	1.1 [0.9, 1.3]
**Contraceptive demand**			
Unmet need	3.3 [3.1, 3.6]	25.8 [23.9, 27.7]	0.6 [0.5, 0.7]
Met need	3.9 [3.7, 4.0]	20.0 [19.2, 20.8]	0.8 [0.7, 0.8]
No demand	7.9 [7.6, 8.2]	26.7 [25.6, 27.8]	2.0 [1.8, 2.2]
**Number of living children**			
No children	19.2 [18.4, 20.1]	39.3 [37.6, 41.0]	6.3 [5.6, 7.0]
1–3 children	3.7 [3.6, 3.8]	20.1 [19.4, 20.8]	0.7 [0.7, 0.8]
4 and above children	3.5 [3.2, 3.9]	16.5 [15.0, 18.1]	0.8 [0.6, 1.0]
**Sex of last child**			
Male	3.6 [3.4, 3.7]	18.5 [17.7, 19.3]	0.7 [0.6, 0.7]
Female	3.8 [3.7, 4.0]	21.3 [20.3, 22.4]	0.8 [0.7, 0.9]
**Regions**			
East	4.9 [4.7, 5.2]	23.8 [22.4, 25.2]	1.2 [1.0, 1.3]
West	4.4 [3.9, 4.8]	21.3 [19.0, 23.6]	0.9 [0.7, 1.2]
North	5.6 [5.3, 5.9]	25.7 [24.4, 27.0]	1.0 [0.9, 1.1]
South	3.9 [3.6, 4.2]	18.9 [17.5, 20.4]	0.9 [0.7, 1.0]
Central	5.6 [5.3, 5.8]	25.5 [24.4, 26.6]	1.1 [1.0, 1.3]
North east	4.1 [3.8, 4.5]	17.7 [16.3, 19.1]	1.1 [0.9, 1.3]
**Total**	**4.9 [4.8, 5.0]**	**23.1 [22.5, 23.8]**	**1.1 [1.0, 1.1]**

Source: National Family Health Survey (NFHS) 2019-21, India

Furthermore, the prevalence of miscarriage during the first trimester (≤12 weeks) was higher among women under the age of 20, currently married, practising the Hindu religion, urban residents, and having more than ten years of education. Miscarriage was also found to be higher among women in the richest wealth quantile, those with no children, and those in the northern and central regions. A higher prevalence of second & above trimesters (>12 weeks) miscarriage is observed among women aged 30 and above, those not currently in a marital union, belong to the Scheduled Castes (SCs) group, lack formal education, fall into the poorest socio-economic category, express a desire for more children in the future, not currently use contraception, having no previous children, having a female child as their last child, and residing in the eastern region.

**Fig. 2:**
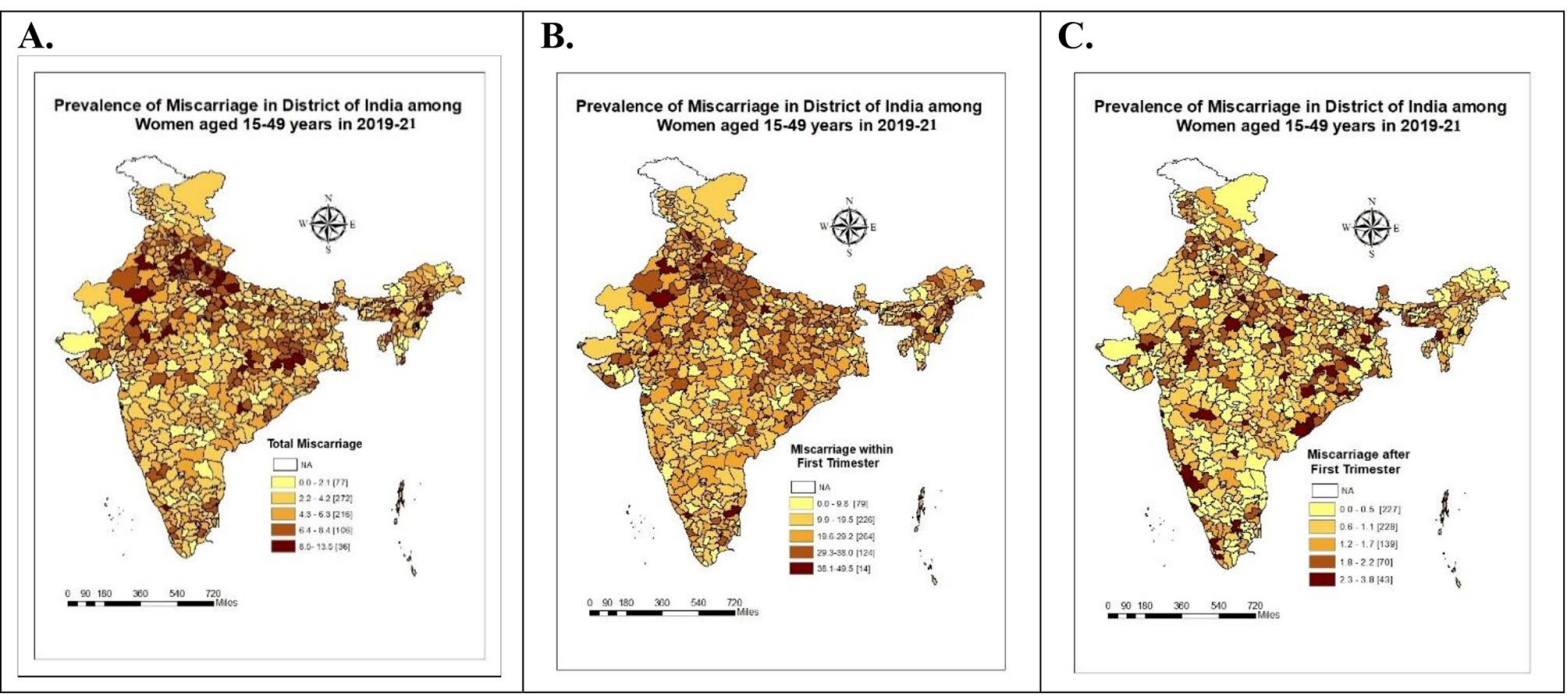
Distribution of prevalence of gestational miscarriage in the first (≤12 weeks) and second & above trimesters (>12 weeks) in the districts of India, NFHS, 2019-21. (Source: National Family Health Survey (NFHS) 2019-21, India)

Figure 2 illustrates the geographical pattern of miscarriage prevalence in the first (≤12 weeks) and second & above (>12 weeks) trimesters across India’s 712 districts. The highest prevalence of miscarriages was reported in the Dhemaji district in Assam (15%), followed by four districts in Manipur and four in Uttar Pradesh, where the prevalence ranges between 8.5% and 13%. First-trimester miscarriages are more common in 31 northeastern and northern Indian districts, whereas second & above trimesters miscarriages were higher in 17 districts, primarily located in the southern and eastern states of the country.

Table 3 presents the results of a generalised linear model with a binomial distribution assumption and a log link for binary outcomes of miscarriage in the first (≤12 weeks) and second & above (> 12 weeks) trimesters. The analysis revealed that women aged 30 years and older have a 9.52 times higher odds ratio (OR: 9.52, 95% CI: 7.34–12.34) for experiencing a miscarriage compared to the reference group of women under 20. Meanwhile, those in the 20–29 age group had 3.47 times higher odds (OR: 3.47, 95% CI: 2.69–4.48) than the reference category. Religious affiliation was also a significant factor, with Hindu women having 1.25 times higher odds of experiencing a miscarriage and women of other religions having 1.28 times higher odds of experiencing a miscarriage than Muslim women. Urban women had a 20% higher likelihood (OR: 1.20, 95% CI: 1.13–1.28) of experiencing a miscarriage compared to rural counterparts. Women with more than ten years of education had a 16% lower likelihood (OR: 0.84, 95% CI: 0.77–0.91) of experiencing a miscarriage compared to uneducated women.

**Table 3:** Generalised linear model with binomial distribution assumption and log link for miscarriage in the first (≤12 weeks) and the second & above (> 12 weeks) trimesters among women in India, NFHS, 2019–21

Variables	Miscarriage		
	Total Pregnancy	First trimester (≤12 weeks)	Second & above trimesters (> 12 weeks)
	Odds Ratio (OR): 95%CI	Odds Ratio (OR):95% CI	Odds Ratio (OR):95% CI
**Age Group**			
<20 years	Ref.	Ref.	Ref.
20–29 years	3.47*** [2.69,4.48]	0.98 [0.71,1.36]	5.35*** [2.75,10.38]
≥30 years	9.52*** [7.34,12.34]	1.15 [0.83,1.61]	19.24*** [9.84,37.62]
**Marital status**			
Currently married	Ref.	Ref.	Ref.
Not in union	0.91 [0.73,1.12]	1.08 [0.84,1.38]	0.92 [0.58,1.47]
**Religion**			
Muslim	Ref.	Ref.	Ref.
Hindu	1.25*** [1.16,1.35]	1.12* [1.02,1.22]	1.18 [0.99,1.41]
Others	1.28*** [1.14,1.44]	1.30*** [1.13,1.49]	0.97 [0.74,1.28]
**Place of residence**			
Rural	Ref.	Ref.	Ref.
Urban	1.20*** [1.13,1.28]	1.10* [1.02,1.19]	1.14 [0.98,1.33]
**Caste**			
Scheduled Tribes (STs)	Ref.	Ref.	Ref.
Scheduled Castes (SCs)	1.37*** [1.25,1.50]	1.26*** [1.13,1.41]	1.24* [1.01,1.51]
Other Backward Class (OBC)	1.44*** [1.32,1.57]	1.38*** [1.24,1.53]	1.14 [0.94,1.38]
Others	1.54*** [1.40,1.69]	1.28*** [1.15,1.44]	1.38** [1.12,1.69]
**Education in years**			
No education	Ref.	Ref.	Ref.
>10 years	1.06 [0.99,1.14]	1.03 [0.94,1.12]	0.99 [0.85,1.15]
≤10 years	0.84*** [0.77,0.91]	0.95 [0.85,1.06]	0.73** [0.60,0.90]
**Wealth Index**			
Poorest	Ref.	Ref.	Ref.
Poorer	1.22*** [1.13,1.32]	1.10* [1.00,1.21]	1.06 [0.90,1.26]
Middle	1.32*** [1.22,1.44]	1.15** [1.04,1.27]	1.16 [0.96,1.39]
Richer	1.30*** [1.19,1.43]	1.12 [1.00,1.25]	1.01 [0.82,1.25]
Richest	1.31*** [1.18,1.46]	1.14* [1.00,1.30]	0.82 [0.63,1.07]
**Desire of children**			
Sterilized/Infecund	Ref.	Ref.	Ref.
Yes	2.81*** [2.58,3.07]	5.70*** [5.12,6.34]	2.15*** [1.78,2.60]
Undecided or no more	1.59*** [1.47,1.73]	2.15*** [1.95,2.38]	1.04 [0.86,1.25]
**Mass media exposure**			
Low	Ref.	Ref.	Ref.
Moderate	1.11*** [1.05,1.18]	1.01 [0.94,1.09]	1.18* [1.01,1.36]
High	1.05 [0.96,1.15]	1.01 [0.91,1.12]	0.99 [0.80,1.24]
**Age at Marriage**			
≥25 years	Ref.	Ref.	Ref.
<18 years	2.66*** [2.40,2.94]	1.18* [1.04,1.33]	3.52*** [2.77,4.46]
18–24 years	1.76*** [1.61,1.93]	1.1 [0.99,1.23]	1.95*** [1.57,2.43]
**Contraceptive Demand**			
Unmet need	Ref.	Ref.	Ref.
Met need	1.27*** [1.18,1.37]	0.87** [0.79,0.96]	1.41*** [1.16,1.70]
No demand	1.46*** [1.34,1.58]	0.53*** [0.47,0.58]	2.14*** [1.75,2.61]
**Number of living children**			
No children	Ref.	Ref.	Ref.
1–3 child	0.56*** [0.46,0.68]	0.76* [0.58,0.99]	0.39*** [0.28,0.56]
4 and above children	0.34*** [0.28,0.42]	0.68** [0.51,0.90]	0.19*** [0.12,0.28]
**Sex of last child**			
Male	Ref.	Ref.	Ref.
Female	0.95* [0.90,1.00]	1.06 [1.00,1.12]	1.05 [0.94,1.18]
**Region**			
East	Ref.	Ref.	Ref.
West	0.63*** [0.56,0.70]	0.70*** [0.61,0.80]	0.76* [0.59,0.98]
North	0.94 [0.87,1.02]	0.96 [0.87,1.06]	1.12 [0.93,1.36]
South	0.55*** [0.50,0.61]	0.58*** [0.51,0.66]	0.68** [0.53,0.86]
Central	0.96 [0.90,1.03]	1 [0.91,1.09]	1.02 [0.86,1.21]
North east	0.88** [0.80,0.97]	0.69*** [0.61,0.77]	0.95 [0.76,1.19]

Source: National Family Health Survey (NFHS) 2019-21, India, India; CI = Confidence Interval in square bracket; Ref = Reference * *p* < 0.05, ** *p* < 0.01, *** *p* < 0.001

Furthermore, women who reported meeting their contraception needs had 1.27 times higher odds of experiencing a miscarriage compared to those who did not report meeting their contraception needs. Across all other regions of the country, women had a lower likelihood of experiencing a miscarriage compared to those residing in the eastern regions. Moreover, the analysis found that women in all other regions of the country had a lower likelihood of experiencing a miscarriage compared to those in the eastern regions. Additionally, we also found that as the number of living children increased, the likelihood of experiencing a miscarriage decreased, particularly in comparison to women with no living children.

During the first trimester (≤12 weeks) of pregnancy, the study found that women who were 30 years of age or older, not in a marital union, had a religion other than Muslim, reside in urban areas, belong to Scheduled Castes (SC), Other Backward Classes (OBC), and other religions groups, expressed a desire for additional children, and were married before the age of 18 were more likely to experience a miscarriage. It was also observed that women who reported meeting their contraception needs or had no demand for contraception faced a lower risk of miscarriage. Furthermore, the study found that as the number of living children increased, the likelihood of experiencing a miscarriage decreased. In the second & above trimesters (>12 weeks), the result indicated that women aged 20 or older, belong to Scheduled Castes (SC) and other religious groups, marrying before the age of 25, desire more children, and either have met their contraception needs or have no demand for contraception had a higher likelihood of experiencing a miscarriage. Appendix Table A1 provide further insights, revealing that overall, women residing in urban areas had a 25% higher likelihood of experiencing a miscarriage compared to their rural counterparts. Notably, when specifically considering first-trimester miscarriages, this likelihood increased to 49% for urban residents compared to rural residents.

Additionally, the analysis reveals that as the wealth status of women improved, their odds of experiencing a miscarriage also increased in comparison to women in the poorest category. In most instances, the interaction effect between wealth status and place of residence was not statistically significant. However, for first-trimester miscarriages, it was observed that individuals in the richest category had 33% lower odds of experiencing a miscarriage compared to those in the poorest category. Interestingly, this effect is modified by the factor of urban residence.

## Discussion

The current study examined the prevalence and risk factors associated with miscarriage among women aged 15–49, considering the occurrence of miscarriage at or after the 12-week mark of pregnancy. We conducted our analysis using the most recent National Family Health Survey (NFHS) 2019–21 calendar data to explore the factors influencing miscarriages during both the first (≤12 weeks) and second & above (> 12 weeks) trimesters of pregnancy. By examining this full spectrum, we comprehensively understood the complexities surrounding pregnant women’s experiences. Our findings underscored the significant impact of various socio-demographic characteristics on the likelihood of miscarriage among reproductive-aged women. These included the mother’s age, urban setting, education level, household wealth status, and geographical region. Notably, older women, women with less than ten years of education, limited exposure to media, unmet contraceptive needs, and no exposed desire for contraception, were at a higher risk of experiencing miscarriage during the second & above trimesters (>12 weeks).

These results align with previous studies carried out in India [6, 38–40], which have demonstrated significantly the relationships between maternal age, education, and miscarriage using NFHS data. Earlier studies using NFHS data have also revealed a higher rate of miscarriage among pregnancies that resulted in miscarriage, with maternal age, education, wealth index, and the place of delivery emerging as potential contributing factors [41].

Our bivariate results found that the prevalence of miscarriage is higher in urban areas of India, which is also consistent with the generalized regression results. These results are consistent with earlier research carried out in Bangladesh and other African nations. [42, 43].

The encouragement of sedentary lifestyles and exposure to pollution, which negatively impact a mother’s health, are potential causes of pregnancy loss in metropolitan settings [44]. Women who want more children tend to have shorter interpregnancy intervals, which could significantly contribute to miscarriages [11]. Many women in metropolitan areas seek to become pregnant later in their reproductive life, which may raise the risk of miscarriage. Women living in urban areas are also often subjected to air pollution, which may negatively impact their pregnancies [45]. The stress and way of life that come with urban living are also known to be significant risk factors for miscarriages in metropolitan women.

After controlling for socioeconomic characteristics, we also discovered contradictory findings on the educational disparities in first (≤12 weeks) and second & above trimesters (>12 weeks) of miscarriages. By using descriptive analysis, we were able to determine that women who had completed ten years of schooling or more had a higher overall and first trimester (≤12 weeks) miscarriage prevalence. On the other hand, women without any schooling have a higher rate of miscarriages in the second & above (> 12 weeks) trimesters. An essential link between educational level and women’s health has already been shown in studies conducted in India [46–48]. In addition, studies also have shown a significant relationship between women’s educational background and their knowledge of concerns relating to pregnancy and reproductive health [49, 50]. The age at which women marry and have children also tends to be postponed by educational achievement. Our results are consistent with those of other studies in this domain. Lower levels of education among women were consistently linked to greater miscarriage rates, according to a hospital-based study conducted in three African nations [51, 52]. They also found that women with only primary education had higher odds of miscarriage than those without formal education, while women with higher education showed similar trends. Other studies differ from this result, which found that the educational levels of the women are not associated with first-trimester miscarriage [28].

In addition, according to our findings, first-trimester miscarriages are more common in women under the age of 20 who are already married. Teenage pregnancy risks the health of both the mother and the child since adolescent mothers typically have lower levels of education. Additionally, teenagers who are pregnant and young mothers frequently lack basic information about pregnancy and child health. Findings from other nations [23, 53] are consistent with these results. A study in Norway has revealed that women aged 25 to 29 have a decreased chance of miscarriage than those aged 30 and older [12]. According to [23], women in their late 30s or older are also at a higher risk of miscarriage, regardless of their reproductive history. Paternal age and maternal age are risk factors for miscarriage; results of a multicenter European study [54].

According to our research, the first trimester and overall miscarriage rates were lower for women in the lowest wealth quintile than for those in the higher quintiles. Additionally, the regression results are consistent with this finding. The cost of public healthcare and economic disparity put pregnant women in danger. One systematic review found that women with higher income quantiles are also more likely to lead sedentary lifestyles, which raises the chance of miscarriage in China among higher wealth quantile women [55]. Another study conducted in Ghana, which was found that the opposite results from our results that, the study found that low socioeconomic status is associated with lower access to an use of maternal health care services, risk factors and poorer pregnancy outcomes for mother and child [56].

In comparison to women who had their last female child, those who had their last male child had a reduced miscarriage rate, according to our descriptive analysis. This outcome, meanwhile, does not align with the regression analysis. These results are consistent with those of earlier research that employed information from telephone interviews and registers to discover that women who had a male firstborn had a significantly higher number of miscarriages than those who had a female firstborn [57]. It was determined that the mother’s vaccination against male-specific minor histocompatibility antigens could potentially be the cause of this [57]. A prospective cohort study and a retrospective analysis discovered that a significant negative predictive factor for women experiencing recurrent miscarriages after having their firstborn child is the male sex of that kid [58]. A study with a limited sample size also discovered that there is no statistically significant correlation between recurrent miscarriage and the prior male and female child [59].

The age of marriage appears to be a significant predictor of miscarriages, according to our regression analysis. The risk of miscarriage is higher for women under the age of 18 who marry than for those who are older than 25. The women who married young had disadvantages with various reproductive health outcomes, such as miscarriage and stillbirth, according to a prior study [60]. Furthermore, a different study discovered that women who marry at the age of 14 or younger experience a higher risk of pregnancy complications such as miscarriage than do women who marry later in life [61]. Our findings also support research from other sub-Saharan African nations, such as Nigeria, Niger, Guinea, Chad, and Mali, which discovered that women who married before the legal age had a 1.27-fold higher lifetime risk of lifelong miscarriages than those who married after the age of eighteen [62]. Even these findings align with research conducted in South Asian nations using sizable sample sizes [63].

Our study also found that regional differences in miscarriage rates. The state of Assam has some of the highest miscarriage rates, followed by Manipur and Uttar Pradesh. However, second & above trimesters (>12 weeks) miscarriages are more frequent in the districts of India’s southern and eastern regions due to poor access to basic healthcare facilities in these areas. Similar results have been reported from a study carried out in Bihar, with a greater miscarriage rate of 8.6% being linked to insufficient maternal and child health initiatives [64]. Women’s antenatal care may also be negatively impacted by growing healthcare disparities among Indian states and rising out-of-pocket costs [65]. Another element that may have a negative impact on pregnancy outcomes is household pollution from the use of cooking fuel. Additionally, many teenagers in developing countries become pregnant, negatively impacting their health and raising the risk of miscarriage.[9]

A greater family size was found to be concurrently connected to miscarriage in our study. Women who previously gave birth alive after experiencing a miscarriage endured pre-eclampsia, threatened miscarriage, underwent induced labour, had an instrumental delivery, had birth prematurely, and had low birth weights [66]. These findings also align with a previous study conducted in India using data from the National Family Health Survey (1998–1999), which found that birth order and age significantly impacted the chance of miscarriage [40].

The investigation of the association between sociodemographic characteristics and miscarriage using a sizable and nationally representative dataset is one of the study’s major strong points. As a result, the accuracy of the conclusions is supported by the statistical analysis of the study’s substantial sample. Notwithstanding this benefit, it is critical to acknowledge the limitations of this research. First of all, this study only used cross-sectional survey data, which made determining causal linkages difficult. Second, there’s a possibility that certain data were over- or underreported because the study relied on self-reported data, which is sensitive to recall bias. Third, these constraints can be addressed in future research by utilising a longitudinal design and an objective measurement technique.

## Conclusions

In our study, women aged 30–39 years, women with lower education, as well as urban women should be the focus of efforts aimed at reducing miscarriages. If the government could have enhanced its general maternal health programmes and focused on the identified group of women, it would be very beneficial. Similar to this, miscarriage women in Indian districts can be divided into these groups and tailored interventions can be arranged through mass media platforms like television to reach these individuals. Public-private health institutions working together can help lower the high rate of miscarriages that is currently affecting the country. Government efforts to reduce the number of miscarriages in India must therefore concentrate on the differences in women’s sociodemographic characteristics.

### Electronic supplementary material

Below is the link to the electronic supplementary material.

Supplementary Material 1

## Data Availability

The dataset is freely available for download at https://dhsprogram.com/data/available-datasets.cfm.

## Abbreviations

DHS: Demographic and Health Survey
NFHS: National Family Health Survey
OR: Odds Ratio
CI: Confidence Interval
