# Supplementary material for: Understanding trimester-specific miscarriage risk in Indian women: insights from the calendar data of National Family Health Survey (NFHS-5) 2019-21
Source: BMC Womens Health. 2024 Jan 23;24:63. doi: 10.1186/s12905-023-02838-7 (PMC10804530; doi:10.1186/s12905-023-02838-7)
Supplement: Supplementary file 1 — Supplementary Material 1 [file 12905_2023_2838_MOESM1_ESM.docx]

**Appendix Table A1:** Estimates of generalized linear model (GLM) for trimesters specific miscarriage: Interaction Effect Analysis

| **Variables** | **Miscarriage** | | |
| --- | --- | --- | --- |
|  | **Total** | **First trimester (**≤**12 weeks)** | **Second & above trimesters (>12 weeks)** |
| **Place of Residence** |  |  |  |
| Rural |  |  |  |
| Urban | 1.25* (1.01, 1.56) | 1.49** (1.13, 1.95) | 0..91 (0.54, 1.52) |
| **Wealth Index** |  |  |  |
| Poorest |  |  |  |
| Poorer | 1.15*** (1.08, 1.23) | 1.11* (1.02, 1.20) | 1.00 (0.87, 1.14) |
| Middle | 1.27*** (1.19, 1.35) | 1.21*** (1.11, 1.31) | 1.02 (0.88, 1.18) |
| Richer | 1.23*** (1.14, 1.33) | 1.23*** (1.12, 1.35) | 0.87 (0.73, 1.03) |
| Richest | 1.35*** (1.23, 1.47) | 1.38*** (1.24, 1.54) | 0.70** (0.55, 0.89) |
| **Place of Residence # Wealth Index (Interaction Effect)** |  |  |  |
| Urban # Poorest |  |  |  |
| Urban # Poorer | 1.04 (0.80, 1.35) | 0.87 (0.63, 1.20) | 1.05 (0.56, 1.96) |
| Urban # Middle | 0.96 (0.75, 1.22) | 0.77 (0.57, 1.04) | 1.25 (0.71, 2.21) |
| Urban # Richer | 0.94 (0.74, 1.19) | 0.72* (0.54, 0.97) | 1.31 (0.75, 2.31) |
| Urban # Richest | 0.86 (0.68, 1.10) | 0.67** (0.49, 0.90) | 1.30 (0.72, 2.33) |

**Source**: National Family Health Survey (NFHS) 2019-21, India, India; CI=Confidence Interval in square bracket; Ref=Reference * p<0.05, ** p<0.01, *** p<0.001
